# Design, fabrication and clinical characterization of additively manufactured tantalum hip joint prosthesis

**DOI:** 10.1093/rb/rbae057

**Published:** 2024-06-03

**Authors:** Dewei Zhao, Liangliang Cheng, Faqiang Lu, Xiuzhi Zhang, Jiawei Ying, Xiaowei Wei, Fang Cao, Chunxiao Ran, Guoshuang Zheng, Ge Liu, Pinqiao Yi, Haiyao Wang, Liqun Song, Bin Wu, Lingpeng Liu, Lu Li, Xiaohu Wang, Junlei Li

**Affiliations:** Department of Orthopaedics, Affiliated Zhongshan Hospital of Dalian University, Dalian 116001, China; Department of Orthopaedics, Affiliated Zhongshan Hospital of Dalian University, Dalian 116001, China; Department of Orthopaedics, Affiliated Zhongshan Hospital of Dalian University, Dalian 116001, China; Department of Orthopaedics, Affiliated Zhongshan Hospital of Dalian University, Dalian 116001, China; Department of Orthopaedics, Affiliated Zhongshan Hospital of Dalian University, Dalian 116001, China; Department of Orthopaedics, Affiliated Zhongshan Hospital of Dalian University, Dalian 116001, China; Department of Orthopaedics, Affiliated Zhongshan Hospital of Dalian University, Dalian 116001, China; Department of Orthopaedics, Affiliated Zhongshan Hospital of Dalian University, Dalian 116001, China; Department of Orthopaedics, Affiliated Zhongshan Hospital of Dalian University, Dalian 116001, China; Department of Orthopaedics, Affiliated Zhongshan Hospital of Dalian University, Dalian 116001, China; Department of Orthopaedics, Affiliated Zhongshan Hospital of Dalian University, Dalian 116001, China; Department of Orthopaedics, Affiliated Zhongshan Hospital of Dalian University, Dalian 116001, China; Department of Orthopaedics, Affiliated Zhongshan Hospital of Dalian University, Dalian 116001, China; Department of Orthopaedics, Affiliated Zhongshan Hospital of Dalian University, Dalian 116001, China; Department of Orthopaedics, Affiliated Zhongshan Hospital of Dalian University, Dalian 116001, China; Department of Orthopaedics, Affiliated Zhongshan Hospital of Dalian University, Dalian 116001, China; Department of Orthopaedics, Affiliated Zhongshan Hospital of Dalian University, Dalian 116001, China; Department of Orthopaedics, Affiliated Zhongshan Hospital of Dalian University, Dalian 116001, China

**Keywords:** selective laser melting, porous Ta, hip joint prosthesis, topology optimization, anti-infection

## Abstract

The joint prosthesis plays a vital role in the outcome of total hip arthroplasty. The key factors that determine the performance of joint prostheses are the materials used and the structural design of the prosthesis. This study aimed to fabricate a porous tantalum (Ta) hip prosthesis using selective laser melting (SLM) technology. The feasibility of SLM Ta use in hip prosthesis was verified by studying its chemical composition, metallographic structure and mechanical properties. *In vitro* experiments proved that SLM Ta exhibited better biological activities in promoting osteogenesis and inhibiting inflammation than SLM Ti6Al4V. Then, the topological optimization design of the femoral stem of the SLM Ta hip prosthesis was carried out by finite element simulation, and the fatigue performance of the optimized prosthesis was tested to verify the biomechanical safety of the prosthesis. A porous Ta acetabulum cup was also designed and fabricated using SLM. Its mechanical properties were then studied. Finally, clinical trials were conducted to verify the clinical efficacy of the SLM Ta hip prosthesis. The porous structure could reduce the weight of the prosthesis and stress shielding and avoid bone resorption around the prosthesis. In addition, anti-infection drugs can also be loaded into the pores for infection treatment. The acetabular cup can be custom-designed based on the severity of bone loss on the acetabular side, and the integrated acetabular cup can repair the acetabular bone defect while achieving the function of the acetabular cup.

## Graphical Abstract

**Figure:**
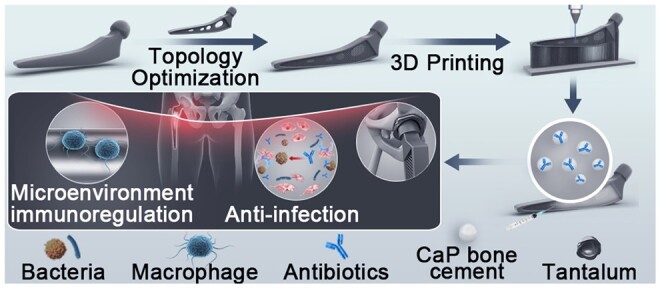

## Introduction

Hip replacement is a common surgical procedure with a high success rate. More than one million total hip replacement surgeries are performed worldwide each year, and this number continues to grow each year [1]. Total hip replacement can effectively relieve pain and improve hip function in patients with advanced arthritis of the hip and significantly improve the quality of life of patients [2]. Hip prostheses, including the femoral stem, acetabular cup and femoral head, are the primary implants used in hip replacement, and this technique has undergone continuous advancements since the mid-century when Wiles, Charnley and other pioneers performed the procedure. Statistics indicate that the lifespan of hip prostheses is generally over 10 years [1]. However, the primary reasons for prosthesis revision include aseptic loosening, prosthesis dislocation, periprosthetic infection, periprosthetic fracture and improper implant positioning [3]. The objectives in optimizing the clinical efficacy of total hip joint prostheses remain the prolongation of their service life, the augmentation of joint function and the minimization of complication rates [4]. To achieve this goal, plasma spraying technology was used to create hydroxyapatite coating at the interface between the lateral prosthesis and bone tissue, forming an osseous binding between the prosthesis and bone tissue and enhancing the osseointegration performance of the implant [5]. In recent years, medical device companies have leveraged thermal spray technology to spray titanium particles on the surface of the femoral stem to obtain rough prosthesis bone contact interfaces [6]. Interestingly, the bone tissue grows into the rough titanium particle interface, forming a mechanical lock between the bone tissue and the prosthesis [7]. Alternatively, a porous tantalum (Ta) interface can be added at the interface between the prosthesis and bone tissue, and the bone tissue can grow into the porous Ta to achieve biological internal fixation, significantly reducing the risk of prosthesis loosening [8]. Joint prostheses are commonly made of metallic materials, given that they are subjected to high loads during service. Upon implantation, a variety of proteins are adsorbed on the surface of the implants, and wear particles are also produced when the implants are rubbed with surrounding tissues or other implants. Non-native proteins adsorbed on the implant's surface recruit Mφ macrophages and induce M1 polarization. The presence of wear particles further promotes M1 polarization. The resulting chronic inflammatory environment causes the implants to be encapsulated by fibrous tissue while activating osteoclasts, ultimately leading to the looseness of the implants. Pro-inflammatory M1 macrophages are eventually replaced by pro-regenerative M2 macrophages during the regeneration process. The prompt transition to M2 macrophages is crucial for successful tissue regeneration. This phenomenon, known as macrophage polarization, refers to the transformation of macrophages into either the pro-inflammatory M1 or pro-regenerative M2 phenotype. In recent years, there has been a growing interest in utilizing immunoregulatory strategies based on material design to enhance osteointegration and extend the lifespan of implants [9].

In addition to material factors, the structural design of the prosthesis plays an important role in its clinical performance. While standardized joint prostheses currently hold a dominant market position, they fail to accommodate the inherent anatomical variations present in individual patients. Moreover, the prevalence of refractory hip diseases, such as joint deformity, prosthesis repair and joint function reconstruction after tumor resection and infection, is increasing; yet, currently available tailored joint prostheses are inadequate [4]. The emergence of additive manufacturing (AM) technology provides a new option for processing and manufacturing technology in many fields [10]. AM technology facilitates the customization of joint prostheses for individual patients. This customization considers the unique joint anatomy of each patient, thereby ensuring an optimal fit between the prosthesis and the native joint. Additionally, AM technology enables the creation of intricate structural features that are challenging to produce using traditional methods. These features can include complex porous structures and intricate internal pore networks within prosthetic components [11]. Among them, the application of micrometer-scale porous structures in bone implants has attracted significant interest [12]. Numerous *in vivo* animal experiments have shown that new bone tissue can grow into porous structures of 200–800 μm [13]. At the same time, the elastic modulus of the porous metal implant can be significantly reduced to a range close to the bone tissue, which can significantly reduce the bone resorption problem caused by stress shielding and achieve the long-term stability of the implant *in vivo* [14]. Furthermore, additive manufacturing technology enables the production of intricate structures that can be integrated without screws or hinges. This enhances the prosthesis’s dependability and minimizes issues such as potential loosening post-implantation [10].

Additive manufacturing offers distinct benefits compared to conventional manufacturing methods, such as rapid design iteration, adaptability to complex geometries and cost efficiency for producing intricate components [15]. Selective laser melting (SLM) is a prevalent technique utilized in the production of metal implants, known for its ability to achieve precise dimensional accuracy and high material density, often with consistent repeatability [16]. Among all kinds of medical metallic materials, Ta has been proven to possess excellent biocompatibility and osseointegration [17]. At the same time, it yields antibacterial and anti-infection effects. Therefore, porous Ta is widely used as orthopedic implant material in clinical practice [18]. This investigation employed SLM to fabricate Ta samples. Subsequently, *in vitro* experiments were conducted to compare the biological properties of SLM-fabricated Ta with those of SLM-fabricated Ti6Al4V. The polarization of RAW 264.7 macrophages on both materials was systematically evaluated by assessing their polarization state and cytokine secretion. Gene expressions and signaling pathways of macrophages cultured on SLM Ta and SLM Ti6Al4V were studied using transcriptomic analysis. Finally, we fabricated a Ta hip joint prosthesis by SLM. The topological structure of the femoral stem was optimized by finite element simulation, and the fatigue resistance of the prosthesis was tested by fatigue test. Meanwhile, porous Ta acetabular cups were designed and printed, and their mechanical properties were tested. Clinical trials were conducted to preliminarily verify the clinical efficacy of porous Ta hip prosthesis fabricated by SLM. In addition, this study investigated the use of porous Ta loaded with calcium phosphate bone cement containing antibiotics as a potential strategy to combat hip infections. Comprehensive evaluations demonstrated that the SLM-fabricated Ta hip joint prosthesis designed in this study exhibited excellent biological performance, mechanical performance and clinical efficacy. These findings highlight the significant potential of our novel approach for clinical application in total hip replacement.

## Materials and methods

### Materials and manufacturing

Spherical Ta powders (purity > 99.95 wt %) from Stardust Technology Co., Ltd were utilized for the production of Ta using SLM technology (Renishaw AM400, UK). The Ta powder was obtained through an inductively coupled plasma spheroidization process. The particle size of Ta powder ranged from 15 to 53 μm. The chemical composition of Ta powder is shown in Table 1 (This data is provided by the vendor).

**Table 1.** Elemental composition of spherical Ta powder

Composition wt %	O	C	N	H	Ti
	0.022	0.0025	0.0024	0.0008	0.001
	Fe	Ni	Mo	Nb	Ta
	0.002	0.001	0.003	0.008	Balance

Sample fabrication was conducted on a high-purity Ta substrate within an inert argon atmosphere containing less than 0.1% oxygen to minimize interstitial oxygen pick-up. Optimized manufacturing parameters were employed in this study, including a laser power of 260 W, a laser scanning point spacing of 50 μm, an exposure time of 100 μs, a layer thickness of 30 μm and a hatch distance of 65 μm. After the printing was completed, the residual stress was eliminated by vacuum heat treatment. The heat treatment process involved a heating rate of 10°C/min, a temperature of 1100°C maintained for a holding time of 2 hours, and a vacuum degree below 10–2 Pa. The printed parts were cooled in the furnace. Spherical Ti6Al4V (Extra Low Impurity, ELI) powders (Stardust Technology Co.) were used as a control group for fabricating Ti6Al4V alloy via SLM using a Renishaw AM400 system (UK). The printing process was conducted in an argon atmosphere with a laser power of 150 W, a laser scanning point spacing of 50 μm, an exposure time of 40 μs, a layer thickness of 30 μm and a hatch distance of 60 μm. Specimens were subsequently wire-electrode cut from the baseplate and heat-treated at 820°C for 1.5 hours in a vacuum environment to relieve internal stress [19].

A Ta cube of 10 × 10 × 10 mm^3^ was printed, and the relative density of the printed Ta cube was measured by the Archimedes method. Samples for OM and SEM were cut from the center of the printed cube and ground with 2000 grit SiC paper, followed by mechanically polishing with 2.5 and 0.5 μm diamond pastes successively. Samples were etched using a solution of 30 ml HCl, 30 ml HF and 15 ml HNO_3_. The microstructure was examined and photographed by an optical microscope to observe the microstructure. The grain sizes were determined by analyzing the optical micrographs with a line-intercept method. Both the planes perpendicular and parallel to the printing direction were observed. Subsequently, the chemical and phase compositions of the SLM-fabricated Ta samples were analyzed using energy-dispersive X-ray spectroscopy (EDS) and X-ray diffraction (XRD). To validate the elemental composition obtained by EDS, inductively coupled plasma optical emission spectrometry (ICP-OES), a carbon-sulfur analyzer and an oxygen-nitrogen-hydrogen analyzer were employed. Tensile testing was performed on an Instron 8872 instrument at room temperature under a strain rate of 10^-^³ s^−1^. Samples were prepared with their geometries parallel to the printing substrate. Five replicates were tested per group.

### Bioactivities of SLM Ta

Mouse preosteoblast cells (MC3T3-E1) were cultured in complete medium (α-MEM medium supplemented with 10% fetal bovine serum, 100 U/ml penicillin and 100 μg/mL streptomycin) in a humidified incubator at 37°C with 5% CO_2_. The medium was changed every 2 days.

#### Cell proliferation and morphology

The samples used in the cell experiments were solid discs with a diameter of 10 mm and a thickness of 3 mm. At 1 and 7 days of culture, the viability of MC3T3-E1 cells on the surface of SLM Ti6Al4V and SLM Ta was assessed by staining living cells with Calcein AM and dead cells with Ethidium Homodimer (LIVE/DEAD cell viability kit, Dojindo, Japan).

The morphology of MC3T3-E1 cells cultured on the surface of SLM Ti6Al4V and SLM Ta was observed by confocal laser microscopy (CLSM). The cells were washed three times with PBS at each defined time point. To prepare samples for CLSM observation, the cells were fixed with 4% paraformaldehyde for 30 min. Then the cells were permeabilized with 0.1% Triton-X 100 in PBS for 5 min. After washing three times with PBS, the cytoskeleton was stained with phalloidin staining solution (1:100, Yeasen) for 30 min. Then, cell nuclei were counterstained with 4’-6-diamidino-2-phenylindole (DAPI) for 5 min. Thereafter, the samples were photographed by CLSM (Zeiss 9000, Germany).

#### Flow cytometry

The MC3T3-E1 cells were seeded on the samples (SLM Ti6Al4V and SLM Ta, discs with a diameter of 20 mm and a thickness of 1 mm) at a density of 2 × 10^6^/well in 6-well culture plates. After 24 hours of culture, the samples were transferred to a new 6-well plate. After washing with PBS, 0.25% trypsin preheated at 37°C was added to digest the cells. After the cells became round, 2 ml of medium was added to prevent digestion. For cell apoptosis measurement, the cells were then resuspended in a 500 μl binding buffer containing 5 μl Annexin V-FITC and 5 μl PI at a concentration of 1 × 10^6^ cells/mL and incubated at room temperature for 15 min. Finally, cell apoptosis was measured by BD flow cytometry. For the measurement of cell cycle distribution, viable cells were collected in a 1.5 ml Eppendorf tube (EP tube) and washed once with PBS. The cell suspension was then centrifuged at 2000 rpm for 5 minutes. Following centrifugation, the cells were resuspended in PBS and adjusted to a concentration of 1 × 10^6^ cells/tube. The cell suspension was again centrifuged at 2000 rpm for 5 minutes. After removal of the supernatant (PBS), the cells were fixed with 500 μl of pre-cooled 70% ethanol overnight at −20°C. Subsequently, the cells were washed twice with PBS and centrifuged at 2000 rpm for 3 minutes. The cells were then stained with 500 μl of a DNA-binding dye solution and incubated in the dark at room temperature for 30 minutes. Finally, red fluorescence intensity was measured using flow cytometry at an excitation wavelength of 488 nm.

#### Cell differentiation

The MC3T3-E1 cells were seeded on the samples (SLM Ti6Al4V and SLM Ta, discs with a diameter of 10 mm and a thickness of 1 mm) at a density of 5 × 10^4^/well and 1 × 10^5^/well in 48-well culture plates. At day 21, the cells were fixed with 4% paraformaldehyde for 10 min, permeabilized by 0.1% Triton-X for 10 min and blocked with 1% goat serum for 30 min. Cells were then incubated with a primary antibody against vinculin (1:500, Abcam) at 4°C overnight and were further incubated with goat anti-mouse secondary antibody (1:1000) at room temperature for 1 h. Cells were stained with 4′6′-diamidino-2-phenylindole (DAPI) to visualize the cell nucleus. Images were subsequently captured using an OLYMPUS fluorescence microscope.

This study investigated the influence of SLM-fabricated Ti6Al4V and SLM-fabricated Ta on the osteogenic differentiation of MC3T3-E1 cells using quantitative real-time polymerase chain reaction (qRT-PCR). MC3T3-E1 cells were seeded at a density of 8 × 10^4^ cells/well onto discs (diameter: 30 mm, thickness: 2 mm) fabricated from either SLM-Ti6Al4V or SLM-Ta and placed in 6-well culture plates. Following cell adhesion, the cultures were maintained in an osteogenic medium, consisting of a complete medium supplemented with 50 mg/L ascorbic acid and 10 mM β-glycerol phosphate. The medium was refreshed every two days. At 14 and 21 days of culture, the expression levels of runt-related transcription factor-2 (Runx2), alkaline phosphatase (ALP), osteocalcin (OCN), osteopontin (OPN) and collagen type-1 (Col-1) were measured to assess the osteogenic differentiation of the MC3T3-E1 cells.

### Macrophage response to SLM Ta and SLM Ti6Al4V *in vitro*

#### Immunofluorescence staining

RAW 264.7 cells were cultured on various samples for 48 hours. Subsequently, the cells were fixed with 4% paraformaldehyde for 20 minutes, permeabilized with 0.25% Triton X-100 for 20 minutes and blocked with 1% bovine serum albumin for 1 hour. To assess the expression of specific markers, the cells were incubated overnight at 4°C with primary antibodies against CD206 (Abcam) and iNOS (Abcam) diluted 1:100. Following primary antibody incubation, the cells were washed and incubated with Alexa Fluor 488 or Alexa Fluor 594 conjugated secondary antibodies for 30 minutes. Finally, the cells were stained with DAPI for 5 minutes for nuclear visualization. A Zeiss LSM 9 confocal laser scanning microscope (Germany) was employed to image the stained cells.

#### Flow cytometry

The expression of surface markers associated with M1 (CCR7) and M2 (CD206) macrophages was evaluated using flow cytometry. RAW 264.7 cells at passage 3 were cultured for 48 hours, harvested by detachment with 0.25% trypsin and centrifuged at 1000 × g for 5 minutes. The supernatant was discarded, and the cell pellet was resuspended in PBS to achieve a density of 1 × 10^6^ cells/100 μl. Cells were then incubated simultaneously with 10 μl each of APC-conjugated CCR7 antibody (Miltenyi Biotec) and PE-conjugated CD206 antibody (BD) for 30 minutes at 4°C in the dark. Following incubation, the cells were washed twice with PBS and resuspended in 0.5 ml PBS for analysis by flow cytometry (FACS Canto II, BD Bioscience, San Jose, CA, USA).

#### Mouse cytokine array panel

RAW 264.7 macrophages were co-cultured with SLM-fabricated Ti6Al4V and SLM-fabricated Ta for 48 hours. To assess cytokine secretion, a cytokine antibody array was employed. Briefly, pre-selected capture antibodies were immobilized onto nitrocellulose membranes. The membranes were then incubated with a mixture of diluted cell lysates and biotinylated detection antibodies specific to the target cytokines. Following incubation, unbound materials were thoroughly washed away. Subsequently, streptavidin conjugated to horseradish peroxidase (HRP) and chemiluminescent detection reagents were sequentially added. The intensity of the light emitted from each spot on the membrane corresponded directly to the amount of bound cytokine, allowing for the quantification of cytokine secretion by the macrophages.

#### Transcriptome sequencing and data analysis

Two milliliters of macrophage cell suspension containing 2 × 10^5^ cells/mL were co-cultured with the various samples in 6-well plates for 48 hours. Subsequently, the macrophages were lysed using Trizol reagent (Beyotime Biotechnology), and the cell lysates were stored at −80°C until RNA sequencing analysis. RNA sequencing was performed on an Illumina NovaSeq 6000 platform (USA). The value of gene expression was transformed as log_10_[FPKM (Fragments Per Kilobase Million) + 1]. Gene Ontology (GO) and Kyoto Encyclopedia of Genes and Genomes (KEGG) pathway enrichment analyses were performed using the free online platform Cloud Platform (https://magic.novogene.com).

#### Differential gene enrichment analysis

The acquired RNA sequencing data was used for Gene Ontology (GO) analysis of differentially expressed genes. This analysis included visualization and statistical evaluation of the functional maps associated with these genes and gene clusters. GO is a comprehensive database that encompasses three ontologies: biological process (BP), cellular component (CC) and molecular function (MF). A hypergeometric test was employed to identify significantly enriched GO terms based on the differential expression patterns of genes within each GO term compared to the entire genome background. Following correction for multiple testing, GO terms with a *P* values less than 0.05 were considered statistically significant.

The Kyoto Encyclopedia of Genes and Genomes is a database that includes the whole genome and all metabolic pathways. It can be used to obtain information regarding the expression levels of specific genes. Therefore, large-scale molecular datasets were generated through genome sequencing and high-throughput databases. The data analysis provides information regarding several advanced functions in different biological systems, such as cells, organisms and ecosystems.

To identify significantly enriched KEGG pathways associated with the differentially expressed genes, this study utilized the ‘Cluster Profiler’ R package for enrichment analysis.

### Topology optimization design, fabrication and performance verification of SLM Ta hip joint prosthesis

#### SLM porous Ta hip stem

The structure of the femoral stem was topologically optimized by finite element simulation. First, the femoral stem model was preprocessed by the software of Ansa to divide the hexahedral mesh. The mesh was imported into Abaqus and loaded with a fixation constraint at the bottom (representing the worst-case scenario for the femoral stem inside the body). Additionally, a load of 2300 N was applied at the femoral head to generate an input file (.inp format). This file was subsequently imported into Tosca software for further analysis. A set named ‘All_Element’ was defined to encompass both the femoral head and the bottom region of the femoral stem. This set was employed to calculate strain energy, volume and other relevant outcomes during the iterative optimization process. To exclude specific areas from optimization, the unit names ‘ASSEMBLY_Head_0’ and ‘ASSEMBLY_SUR_1’ within the .inp file were renamed to ‘Head’ and ‘Surface’, respectively. These names now directly correspond to the solid unit of the femoral head and the bottom surface unit. Throughout the optimization iterations, the volume served as the design response, and the volume value of the ‘All_Element’ set was extracted after each iteration. The strain energy, serving as the design response, was incorporated into the optimization algorithm to guide the removal of elements. The volume, acting as a constraint, limited the extent of material reduction. The optimization process continued iteratively until the target objective was achieved.

The SLM process yielded a porous Ta femoral stem implant that closely resembled the simulation analysis model. Following fabrication, both the SLM femoral stem and its substrate underwent overall heat treatment. Subsequently, the implant surface was polished to achieve a smooth finish by removing surface steps and rough grains. To assess fatigue performance, the femoral stem was then subjected to testing procedures aligned with the requirements outlined in ISO 7206-21 for partial and total hip joint prostheses. During testing, epoxy resin was employed as the embedding medium. The fatigue testing regimen for the femoral stem itself utilized a loading force of 2300 N, a loading frequency of 10 Hz, a stress ratio (*R*) of 0.1 and a total test duration of 5 million cycles. For fatigue testing of the femoral stem’s neck region, a loading force of 5340 N, a loading frequency of 10 Hz, a stress ratio (*R*) of 0.1 and a total test duration of 10 million cycles was implemented.

#### SLM porous Ta acetabular cup

Creo software was used to design the porous Ta acetabulum cup. The cup was then printed onto a Ta substrate, with high-purity argon being used as a protective atmosphere during the printing process. After heat treatment, the acetabulum cup was separated from the substrate using wire cutting, and any remaining Ta powder was removed through sandblasting. This study investigated the deformation behavior of acetabular shells and the disassembly forces required for modular acetabular devices, following the testing methodologies outlined in ISO 7206-12 and ASTM F1820.

### Clinical application of personalized hip implant

#### Study population

This retrospective study included 40 patients who underwent total hip arthroplasty (THA) surgery for the treatment of hip joint disease at our institution between 2019 and 2021. All surgical procedures were conducted in accordance with the ethical principles established by the responsible committee on human experimentation (both institutional and national) and adhered to the tenets of the Declaration of Helsinki as revised in 2008. Written informed consent for study participation was obtained from all patients. This study protocol received approval from the Ethics Committee of the Affiliated Zhongshan Hospital of Dalian University (approval number: ChiCTR-ONC-17012274).

#### Preoperative planning and design

The prostheses used in the 3D printing group were designed by the authors preoperatively and manufactured by the Orthopedic Medical Research Center of Dalian University and Beijing Lidakang Company. First, a thin-slice CT of the hip joint and femur was performed. Next, the corresponding CT data were imported into Mimics 20.0 software to build a 3D model of the affected femur. Based on the femoral marrow cavity's size and shape, an initial model of a customized filling prosthesis was generated. The prosthesis was tailored to match the size of the femoral marrow cavity, and a 3D porous structure was designed at the interface between the prosthesis and the femoral bone to promote bone growth. The prosthesis was subsequently printed using a 3D printer.

#### Surgical method

After anesthesia, the patients were placed in the lateral decubitus position. The skin was routinely disinfected, the anterior-lateral approach was used, and the skin and subcutaneous tissue were cut layer by layer to expose the lesion. The femoral head was cut based on the preoperative design, while in the scenario of a revision total hip arthroplasty, the femoral prosthesis was removed. Following reaming of the femoral canal to the desired dimensions, the femoral prosthesis was carefully inserted. To ensure proper fit and stability, the prosthesis was then impacted (compacted) with a surgical mallet. The stability of the implant was subsequently evaluated. Patients in the control group received the best commercially available filling prosthesis, while those in the 3D-printed group received customized prostheses fabricated using additive manufacturing techniques. Upon completion of all standard surgical procedures, the incision was closed in a layered fashion using sutures.

#### Postoperative treatment and follow-up

Prophylactic intravenous antibiotic administration commenced routinely on postoperative day 1 (POD1). On the first postoperative day, a physical therapy regimen incorporating gentle exercises for the affected lower extremity was initiated. Patients ambulated without weight-bearing using crutches. Weight-bearing progression was determined on an individualized basis, informed by the specific characteristics of the implanted prosthesis. Following confirmation of adequate drainage (volume less than 30 ml), the drainage tube was removed, typically two to three days postoperatively. Patients were followed up at 1, 3, 6 and 12 months postoperatively and once a year. The Harris hip scores (HHS), subjective satisfaction and presence of complications were recorded at follow-ups. The incision length, duration of surgery and intraoperative blood loss were recorded and compared between groups.

### Statistical analysis

Quantitative data were presented as the mean ± standard error (SE). All *in vitro* experiments were performed in triplicate to ensure reproducibility. Statistical analysis was performed using analysis of variance (ANOVA) to identify significant differences between groups. A *P* values of less than 0.05 was considered statistically significant.

## Results

### Fabrication and characterization of SLM Ta

**Figure 1.**
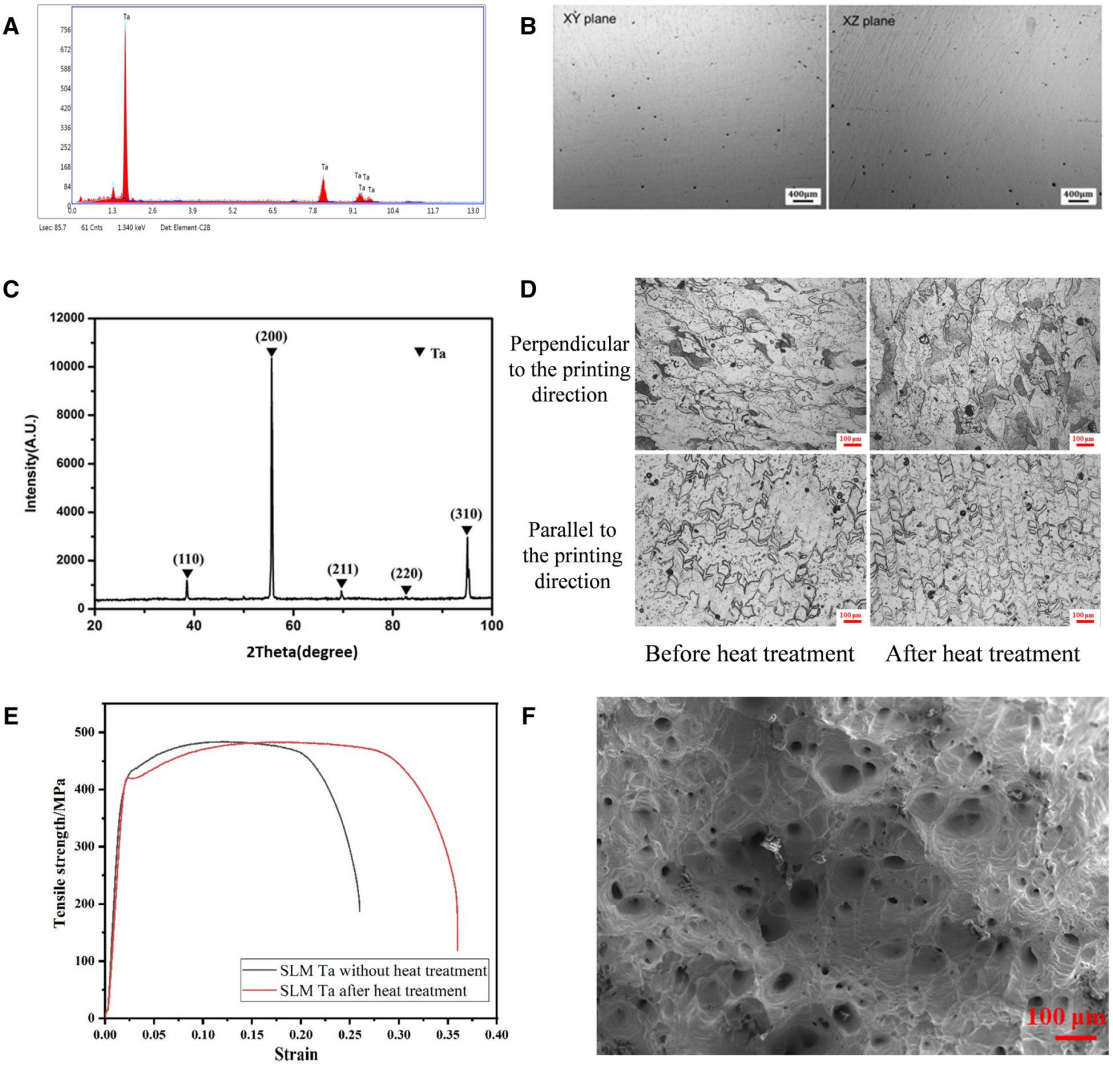
(A) Element components of SLM Ta detected by EDS, (B) internal defect distribution of SLM Ta after polishing, (C) XRD patterns of SLM Ta, (D) metallographic microtopography of SLM Ta, (E) tensile mechanical property curves SLM Ta, (F) fracture morphologies of SLM Ta after tensile test.

Table 2 presents the chemical composition of the SLM-fabricated Ta implant. The oxygen content was measured at 0.024 wt %, indicating minimal change compared to the raw material. Furthermore, the impurity levels in the SLM-fabricated Ta conform to the established standards outlined in ISO 13782 [20]. The EDS analysis results (Figure 1A) were consistent with the ICP analysis results. Ta was the main constituent of the printed parts. Figure 1B shows the XRD patterns of SLM Ta. All characteristic peaks were in good agreement with the JCPDS X-ray powder standards (JCPDS 04-0788). The SLM Ta exhibited a body-centered cubic crystal structure. As shown in Figure 1C, the SLM Ta cube was polished to observe internal defects. Only a few pores were observed inside the SLM Ta. The microstructure morphology of SLM Ta before and after heat treatment was observed by metallographic microscopy (Figure 1D). The grain size of SLM Ta ranged from 60 to 100 μm. Figure 1E shows the tensile mechanical property curve of SLM Ta. Before heat treatment, the yield strength and tensile strength of SLM Ta were 429 MPa and 484 MPa, and the elongation was 26%. After heat treatment, the yield strength and tensile strength of SLM Ta were 420 MPa and 483 MPa, respectively, and the elongation was 36%, indicating that the vacuum heat treatment could significantly improve the plastic deformation ability of SLM Ta without affecting the strength. Figure 1F presents the fracture morphology of SLM-fabricated Ta. The fracture surface exhibited numerous dimples, a characteristic indicative of ductile fracture. Table 3 shows the values of relative density and surface roughness of SLM Ta. The relative density of the SLM Ta cube reached 99.5%, the roughness of the printed surface parallel to the substrate was 2–4 μm, and the roughness of the outer surface perpendicular to the substrate was 9–11 μm.

**Table 2.** Chemical composition of SLM Ta

Chemical composition wt %	O	C	N	H	Si	Ti
	0.024	0.0024	0.0015	0.0005	<0.005	<0.001
	Fe	Ni	Mo	Nb	Mo	Ta
	<0.002	<0.001	<0.003	<0.008	<0.001	Balance

**Table 3.** Properties of SLM Ta

Properties	Value
Relative density measured by the Archimedes method	99.5%
Surface roughness parallel to the printing direction	2–4 μm
Surface roughness perpendicular to the printing direction	9–11 μm

### *In vitro* bioactivity

**Figure 2.**
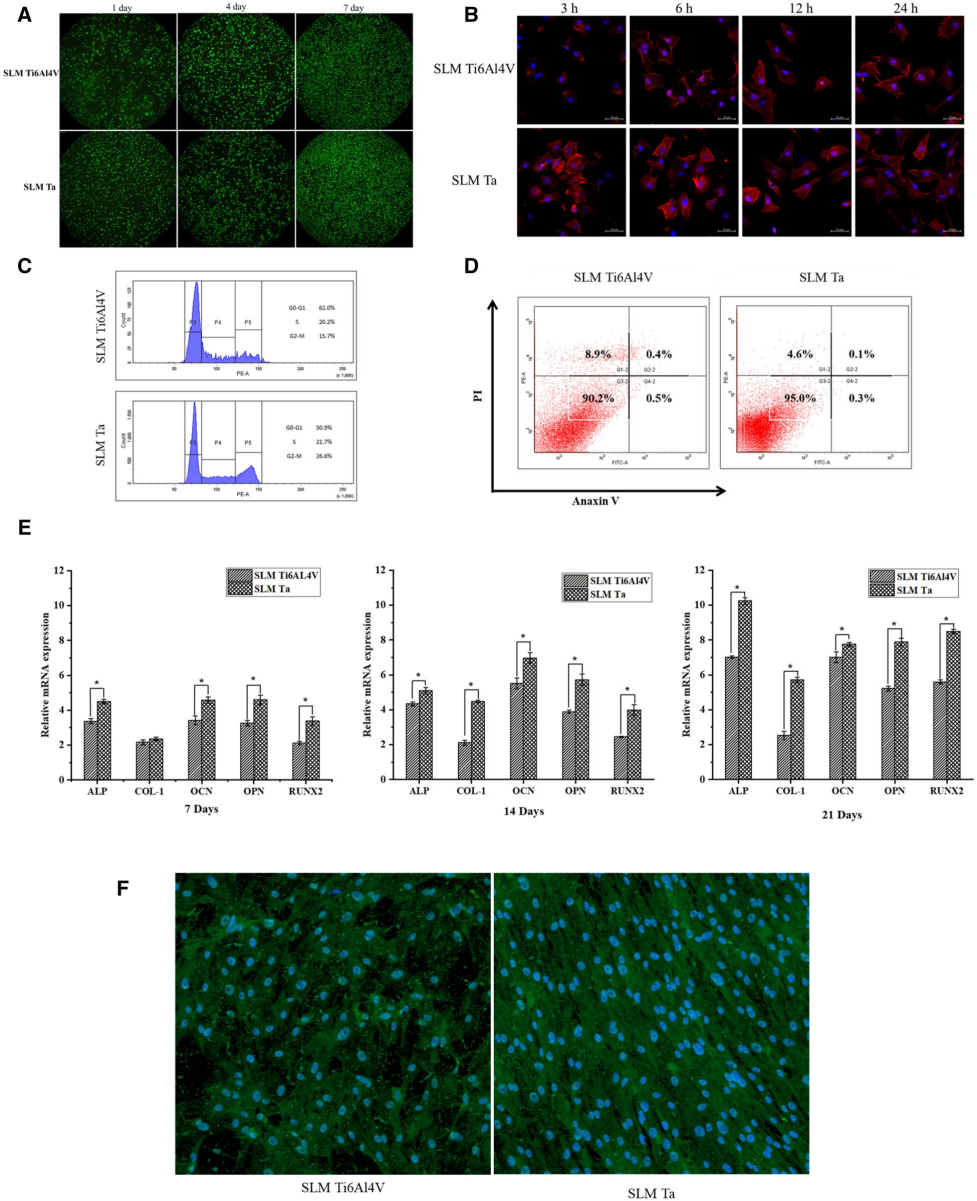
(**A**) Live-dead staining of cells cultured on the surfaces of SLM Ti6Al4V and SLM Ta for 1, 4 and 7 days, (**B**) actin (red) and cell nucleus (blue) fluorescence images of MC3T3-E1 cells cultured on the surfaces of SLM Ti6Al4V and SLM Ta for 3, 6, 12 and 24 h, (**C**) the cell cycle distribution of MC3T3-E1 cells cultured on the surfaces of SLM Ti6Al4V and SLM Ta detected by flow cytometry, (**D**) the apoptosis of MC3T3-E1 cells cultured on the surfaces of SLM Ti6Al4V and SLM Ta detected by flow cytometry, (**E**) expression of ALP, col-1, OCN, OPN and Runx2 measured based on relative mRNA expression after 7, 14 and 21 days of cell culture on the surfaces of SLM Ti6Al4V and SLM Ta and (**F**) ALP activity of MC3T3-E1 cells cultured on the surfaces of SLM Ti6Al4V and SLM Ta for 21 days.

Figure 2A depicts the results of the live/dead staining assay for MC3T3-E1 cells cultured on SLM-fabricated Ti6Al4V and SLM-fabricated Ta surfaces over a period of 1, 4 and 7 days. The findings suggest that cell viability was superior for cultures grown on SLM-Ta surfaces compared to those on SLM-Ti6Al4V surfaces. Furthermore, CLSM was employed to evaluate the initial attachment of MC3T3-E1 cells on various surfaces at 3, 6, 12 and 24 hours. The SLM Ta surface demonstrated a higher number of MC3T3-E1 cells spreading, as displayed in the first column of Figure 2B, compared to the SLM Ti6Al4V surface. Moreover, the extent of cell spreading on the SLM Ta surface was more significant, and the number of cells was higher than in the SLM Ti6Al4V group, consistent with the results obtained from the live/dead staining. These findings suggested that SLM Ta possessed better cell affinity than SLM Ti6Al4V. The number and area of cell spreading on the SLM Ta surface did not vary significantly, implying that stable adhesion of MC3T3-E1 cells to the SLM Ta surface occurred within 3 hours. In contrast, the abundance of cells on the SLM Ti6Al4V surface increased slightly over time, accompanied by increased cell spreading area. Stable cell adhesion was achieved on the SLM Ti6Al4V surface at 12 h. Figure 2C depicts the cell cycle distribution of MC3T3-E1 cells cultured on SLM-fabricated Ti6Al4V and SLM-fabricated Ta, as determined by flow cytometry. This analysis serves as an indicator of cellular proliferation. The percentage of MC3T3-E1 cells undergoing division on the surfaces of SLM-fabricated Ti6Al4V and SLM-fabricated Ta was 15.7% and 22.7%, respectively. Figure 2D illustrates the apoptotic state of MC3T3-E1 cells cultured on the surfaces of SLM-fabricated Ti6Al4V and SLM-fabricated Ta, as assessed by flow cytometry. The PI-positive (nonviable) cell population on SLM-fabricated Ti6Al4V and SLM-fabricated Ta surfaces was 9.3% and 7.4%, respectively. These findings suggest that SLM-fabricated Ta exhibits superior biocompatibility compared to SLM-fabricated Ti6Al4V, corroborating the positive influence of Ta on cell proliferation. Cell differentiation was assessed at 7, 14 and 21 days with osteogenic markers, including ALP, Col-1, OCN, OPN and Runx2 by qRT-PCR tests (Figure 2E). The expression level of osteogenesis-related genes in MC3T3-E1 cells cultured on SLM Ta was significantly higher than the SLM Ti6Al4V group, which proved its superior osteogenic activity. The ALP expression of 5 × 10^4^ MC3T3-E1 cells cultured on SLM Ti6Al4V and SLM Ta surfaces after 21 days was characterized by an immunofluorescence assay (Figure 2F). The ALP expression level of MC3T3-E1 cells cultured on SLM Ta was higher than that of SLM Ti6Al4V, which indicated that SLM Ta could promote ALP expression of MC3T3-E1 cells compared with SLM Ti6Al4V.

### Activation of macrophages on different samples

**Figure 3.**
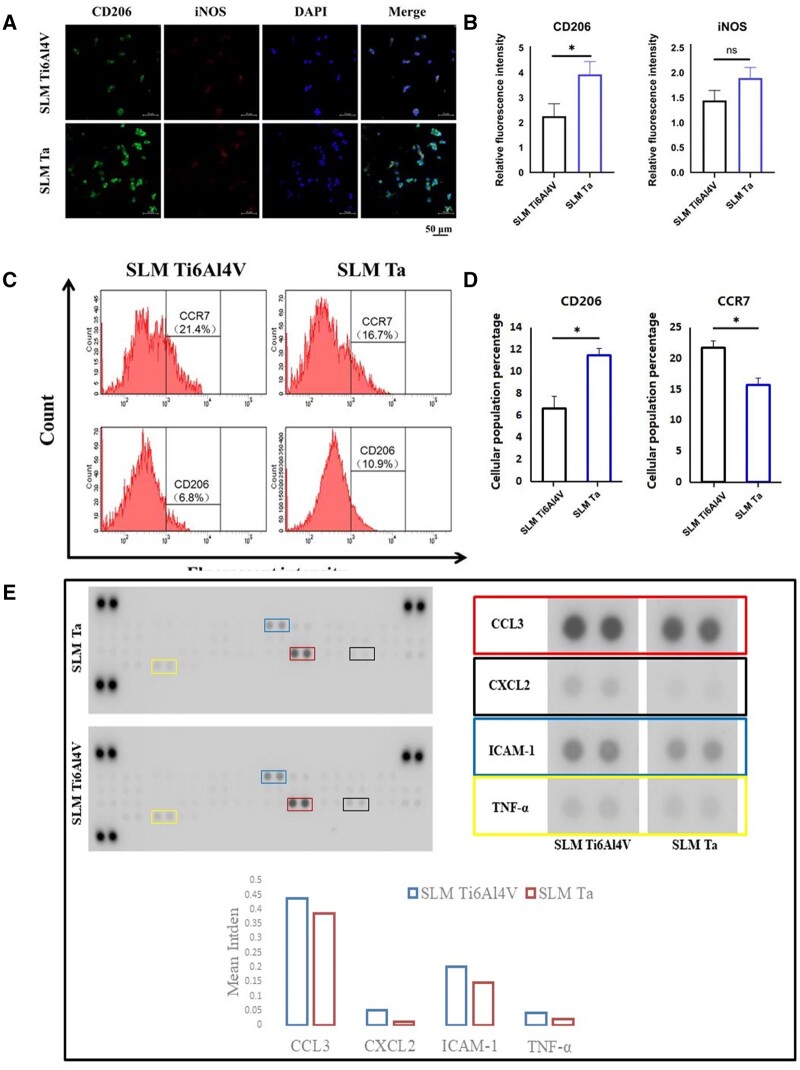
(**A**) Fluorescence microscopy images of CD206, iNOS and nucleic staining of macrophages on SLM Ti6Al4V and SLM Ta, (**B**) quantitative fluorescence intensity of CD206 and iNOS of macrophages on SLM Ti6Al4V and SLM Ta, (**C**) polarization of macrophages was evaluated by the expressions of CCR7 (M1) and CD206 (M2) using flow cytometry, (**D**) quantitative fluorescence intensity of CCR7 and CD206 of macrophages on SLM Ti6Al4V and SLM Ta and (**E**) protein expression levels of inflammatory cytokines in macrophages cultured on SLM TI6AI4V and SLM Ta.

Immunofluorescent staining of macrophages was used to assess the effects of SLM Ti6Al4V and SLM Ta on the expression of iNOS and CD206 (Figure 3A and B). CD206 (M2 macrophage marker) and iNOS (M1 macrophage marker) were selected for investigating macrophage polarization. In the SLM Ti6Al4V and SLM Ta groups, the average fluorescence intensities of iNOS were 1.24 ± 0.22 and 1.89 ± 0.21, respectively. There was no significant difference between the two groups (*P* > 0.05). Compared with the SLM Ti6Al4V group, the expression of CD206 in the SLM Ta group was significantly increased (3.93 ± 0.51, *P* < 0.05), indicating the significantly higher anti-inflammatory response of macrophages in the SLM Ta group.

CD206 is a cell-surface protein highly expressed on M2 macrophages and serves as a distinctive marker for identifying cells with M2 polarization. Conversely, the presence of C-C chemokine receptor type 7 (CCR7) on the surface of a macrophage is a hallmark of the M1 phenotype, making it a useful marker for distinguishing M1 from M2 macrophages. Flow cytometry was used to evaluate the polarization of macrophages to further confirm the phenotype of polarized macrophages on SLM Ti6Al4V and SLM Ta. As shown in Figure 3C and D, the proportion of M1 macrophages in the SLM Ti6Al4V group was 21.4%, while the proportion of M1 macrophages in the SLM Ta group was lower, at 16.7%. Moreover, the proportion of M2 macrophages was 6.8% in the SLM Ti6Al4V group, while in the SLM Ta group, it was higher, at 10.9%. Taken together, these findings suggest that SLM Ta may induce a lower immune inflammatory reaction and rejection after transplantation than SLM Ti6Al4V and may be more conducive to tissue repair facilitated by M2 macrophages. Therefore, compared to SLM Ti6Al4V, SLM Ta may offer advantages for tissue repair, owing to its potential to promote M2 macrophage-mediated healing and reduce immune-inflammatory responses.

In this study, macrophages were cultured on two different materials, SLM Ti6AI4V and SLM Ta, for 48 hours. Next, the macrophages were collected, and their proteins were extracted. The expression levels of these proteins were then analyzed using Mouse Cytokine Array Panel A to compare the effects of the two materials on macrophage protein expression (Figure 3E). Gray value analysis showed that the protein expression levels of macrophage inflammatory chemokine CCL3, CXCL2, TNF-α and cell adhesion factor ICAM-1 significantly differed between SLM Ti6AI4V and SLM Ta groups. Compared with macrophages cultured on SLM Ta, the pro-inflammatory factors were significantly higher expressed in macrophages cultured on SLM Ti6AI4V, indicating that macrophages cultured on SLM Ta showed a relatively weaker inflammatory response.

### Bioinformatic analysis of macrophage gene expression on SLM Ta

**Figure 4.**
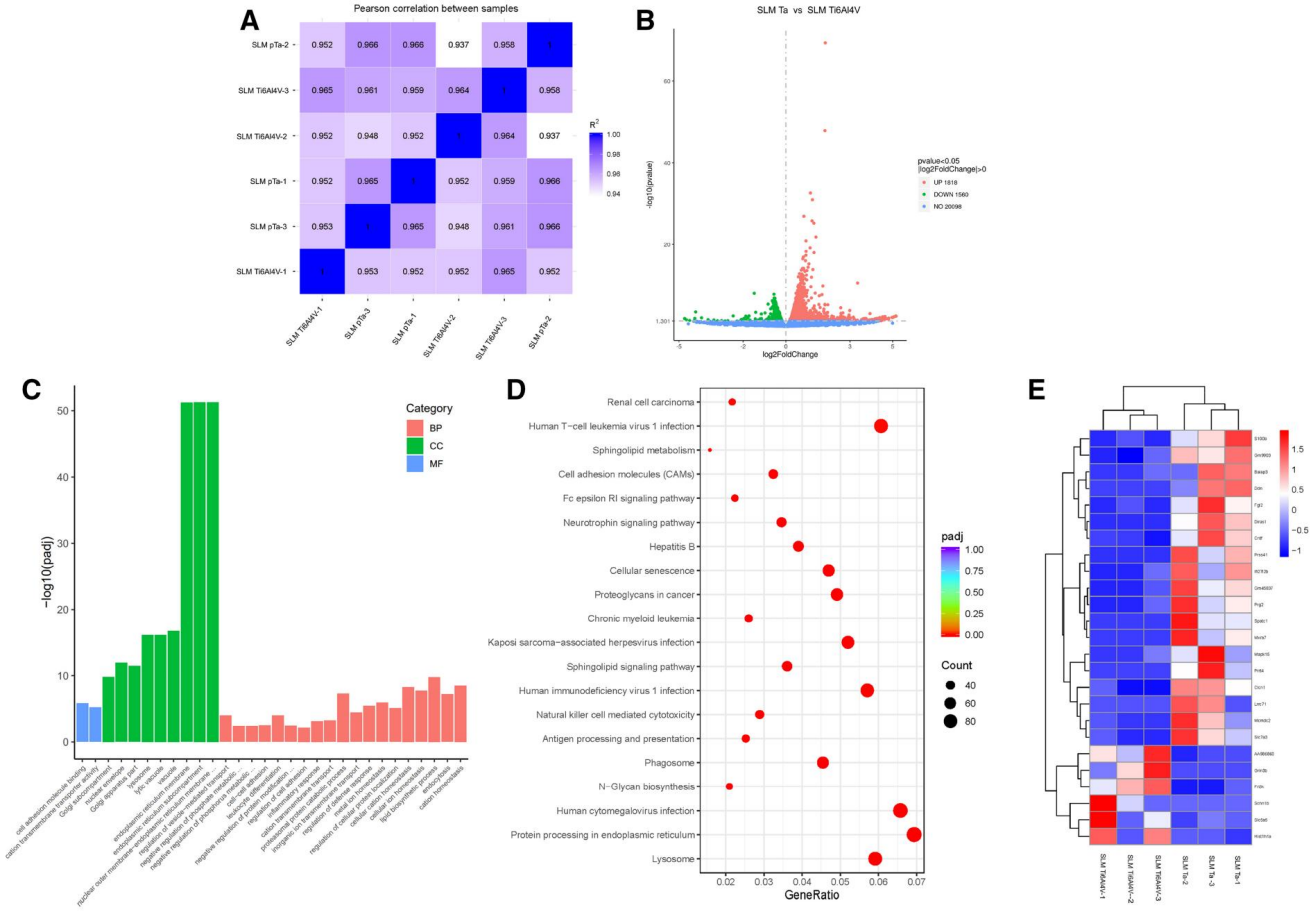
Bioinformatic and mechanistic analysis of macrophage gene expression and polarization induced by SLM Ta and SLM Ti6Al4V. (**A**) Heatmap of Pearson correlation between samples; (**B**) volcano plot of transcriptomic analysis of differentially expressed genes, *n* = 3 independent experiments per group; (**C**) GO analysis of all genes in macrophages cultured on SLM Ta and SLM Ti6Al4V. The three colors, orange, green and blue, are used to represent the three main gene ontology categories, which are biological process, cellular component and molecular function, respectively; (**D**) enriched KEGG pathways of SLM Ta and SLM Ti6Al4V. The abscissa is the ratio of differential genes linked with the KEGG pathway to the total number of differential genes. The ordinate is KEGG pathway. The dot size represents the number of genes annotated to a specific KEGG pathway. The color gradient from red to purple represents the statistical significance of enrichment; (**E**) heatmap analysis of differentially expressed genes (protein_coding |log_2_fold-change|≥3).

To elucidate the mechanisms by which SLM-fabricated Ta and SLM-fabricated Ti6Al4V influence macrophage polarization, transcriptomic analysis of macrophages cultured on these materials was employed. Initially, the Pearson correlation coefficient was calculated between samples to assess their stability through correlation analysis. Most correlation coefficients fell within acceptable ranges, exceeding 0.93 (Figure 4A). Transcriptomic analysis further revealed the number of differentially expressed genes between the two groups with a focus on genes with adjacent expression levels (SLM Ta versus SLM Ti6Al4V). As illustrated in Figure 4B, the volcano plots depicted substantial variation in gene expression, with 1818 genes upregulated and 1560 genes downregulated.

Next, the differentially expressed genes were then subjected to Gene Ontology (GO) analysis (BP, MF, CC) to identify enriched functional categories (top 30 shown in Figure 4C). Similarly, KEGG pathway analysis revealed enriched pathways (top 20 shown in Figure 4D) potentially underlying these differences. Focusing on protein-coding genes, the resulting heatmap (Figure 4E) depicts the expression patterns of DEGs.

### Structural optimization, fabrication and performance verification of SLM Ta hip prosthesis

**Figure 5.**
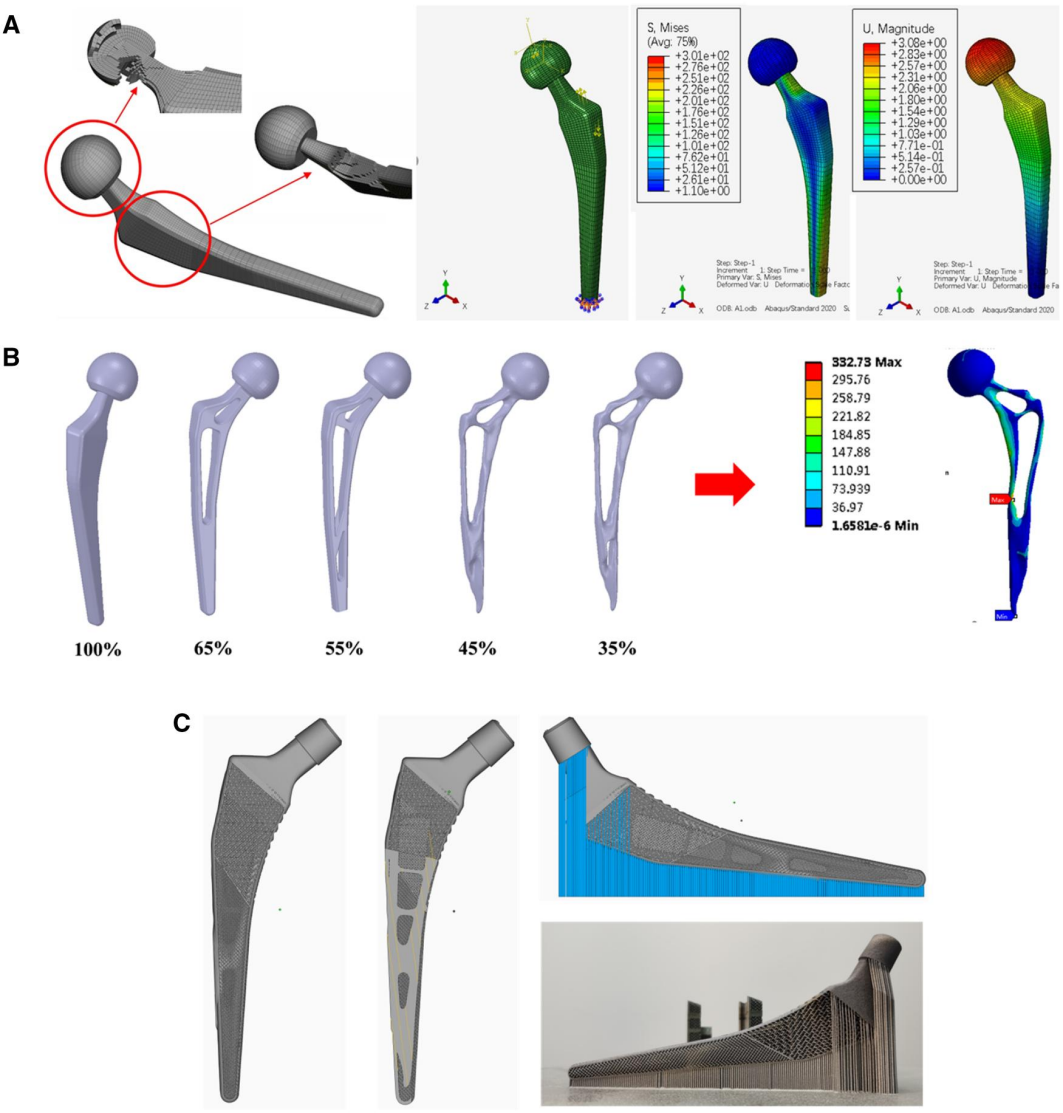
(**A**) Mesh processing, the fixation constraint and loading of SLM Ta femoral stem, strain and stress distribution of femoral stem in physiological load environment calculated by finite element simulation method, (**B**) topological optimization of femoral stem after smoothing with different residual weight and model of Ta femoral stem with porous structure and (**C**) Fabrication of Ta femoral stem with porous structure using SLM technology.

Figure 5A illustrates the intricate mesh details of the femoral stem, the boundary conditions applied during the worst-case scenario simulation, and the resulting stress and strain distribution. Notably, employing hexahedral elements throughout the mesh significantly enhanced the accuracy of the simulation. The maximum stress experienced by the femoral stem was 301 MPa, which falls below the yield strength of SLM-fabricated Ta (420 MPa). The maximum strain was measured at 3.08%. Prosthesis configurations with different volume fractions (65%, 55%, 45%, 35%) were obtained, as shown in Figure 5B. The maximum stress on the optimized femoral stem (with 35% original weight) was 332.73 MPa. Porous Ta was used to supplement the optimized deducted part. The SLM Ta femur stem with the optimized structure was fabricated by selective laser melting (Figure 5C).

**Figure 6.**
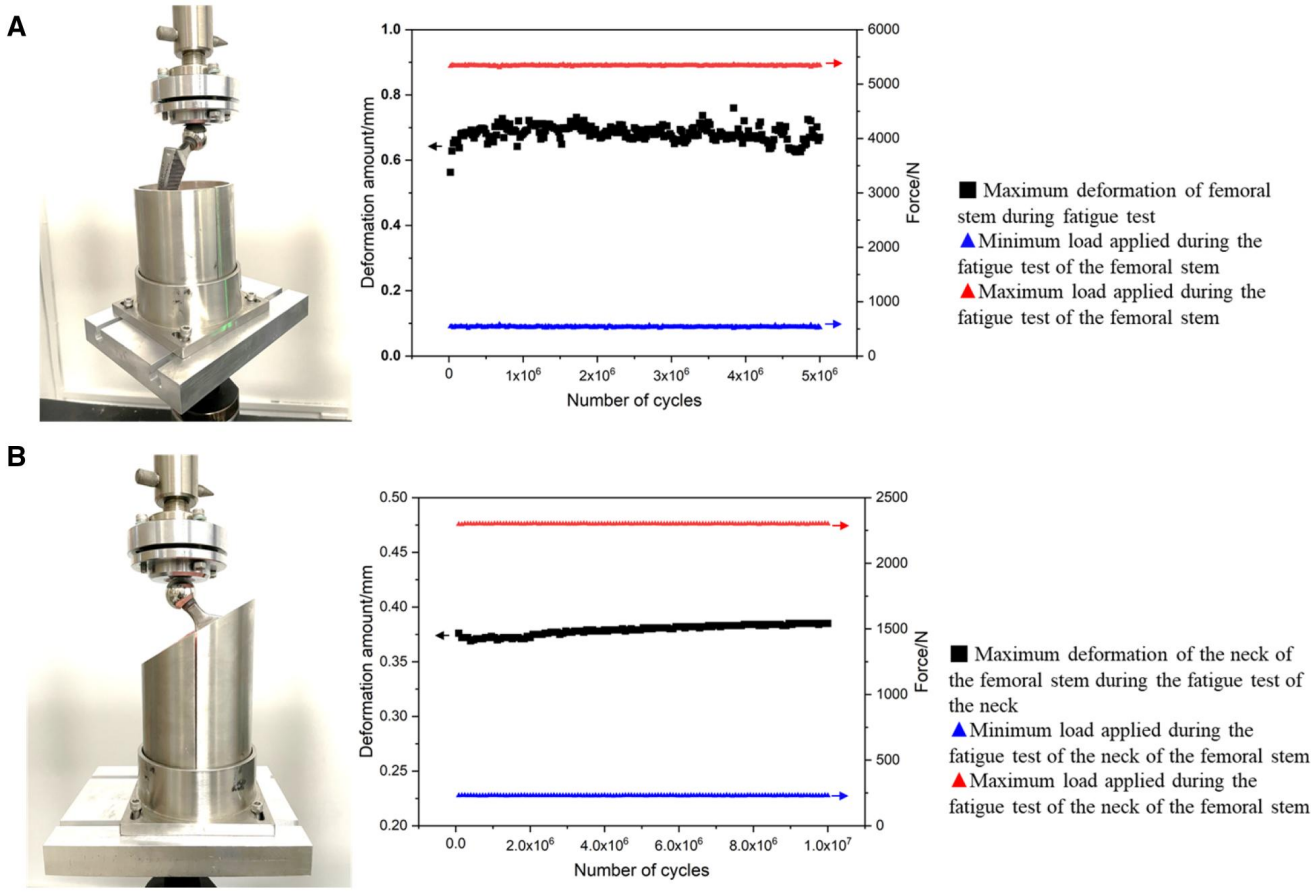
Fatigue test of SLM Ta femoral stem (**A**) and the neck (**B**) of SLM Ta femoral stem.

After heat treatment, grinding and polishing of the femoral stem, the fatigue performance of the femoral stem was tested by the fatigue testing machine. As shown in Figure 6, stable deformation was observed for the femoral stem and the neck of the femoral stem during the fatigue test. During the fatigue test of the femoral stem (Figure 6A), the deformation ranged from 0.6 mm to 0.8 mm. In contrast, the neck of the femoral stem (Figure 6B) exhibited a more consistent deformation, measuring approximately 0.375 mm. After 5 million cycles, no damage was found in the 6 femoral stems. After 10 million cycles, no damage was found in the neck of the femoral stem. Therefore, it can be concluded that the SLM Ta femoral stem, after topology optimization, can meet the requirements of ISO 7206 for the fatigue performance of the femoral stem.

**Figure 7.**
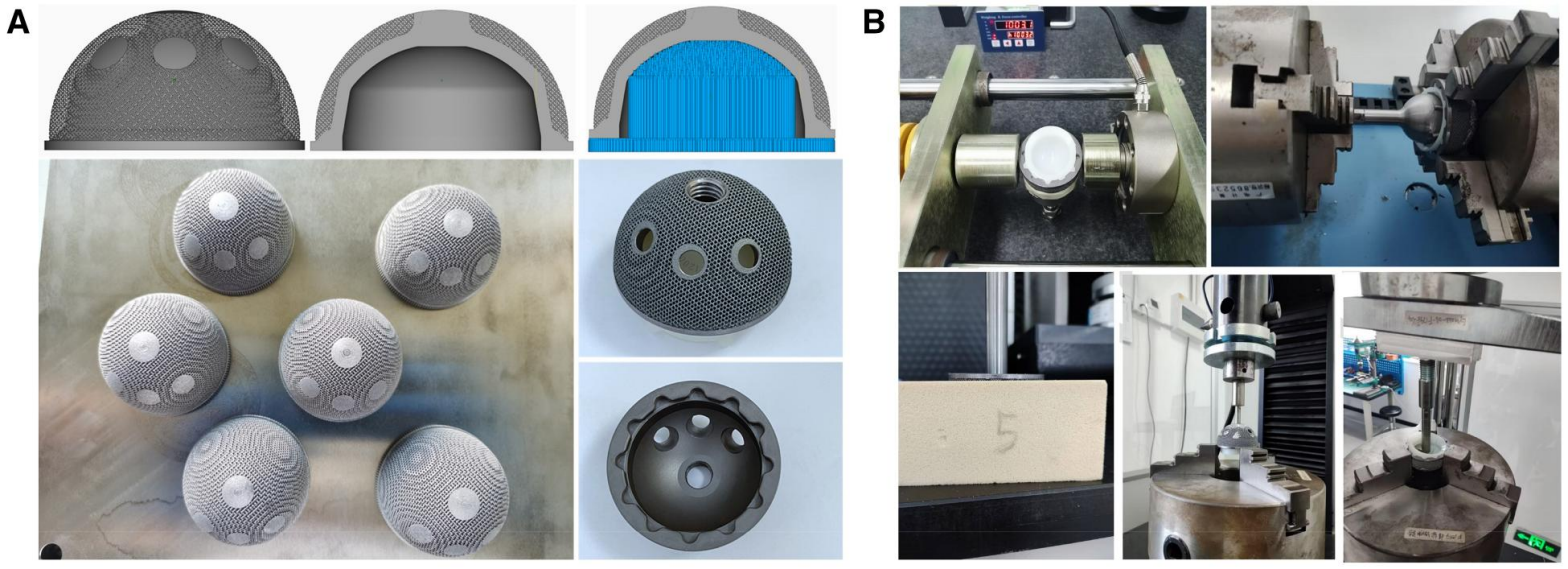
(**A**) Fabrication of porous Ta acetabular cup using SLM technology and (**B**) the performance test of the SLM porous Ta acetabulum cup according to ISO 7206-12 and ASTM F1820.

As shown in Figure 7A, the SLM porous Ta acetabulum cup exhibited a uniform shape and smooth surface. The initial inner diameter *D*_0_ of the acetabular cup was 41.4806 ± 0.0021 mm. The performance of the SLM porous Ta acetabulum cup was tested as shown in Figure 7B. When 1000 N axial force was applied to the acetabular cup, the inner diameter *D*_1_ of the detected acetabular cup was 41.1971 ± 0.0023 mm, and the inner diameter *D*_2_ after unloading was 41.4770 ± 0.0018 mm, (*D*_2_ − *D*_0_)/(*D*_0_ − *D*_1_) = 1.27% < 2%, suggesting that the porous Ta acetabular cup did not undergo plastic deformation. The axial push force, eccentric pull-out force and maximum torsional separation torque for disassembly of the acetabular cup and polyethylene liner were 1371.52 ± 219.04 N, 341.02 ± 34.45 N and 46.04 ± 1.99 N·m, respectively, which were consistent with the commercial acetabular cup. After the impact test, the shape of the acetabular cup did not change, which validated the satisfactory impact resistance performance of the SLM Ta acetabular cup. A slight deformation was observed in the porous layer of the acetabular cup, which helped to enhance the matching between the acetabular cup and the bone tissue at the implant site and improved the friction between the implant and the bone tissue at the initial stage of implantation. At the same time, the close coordination between the porous interface and the bone tissue helped the bone tissue to grow into the porous layer quickly, resulting in biological internal fixation and improving the long-term stability of the acetabular cup.

### Clinical application

**Figure 8.**
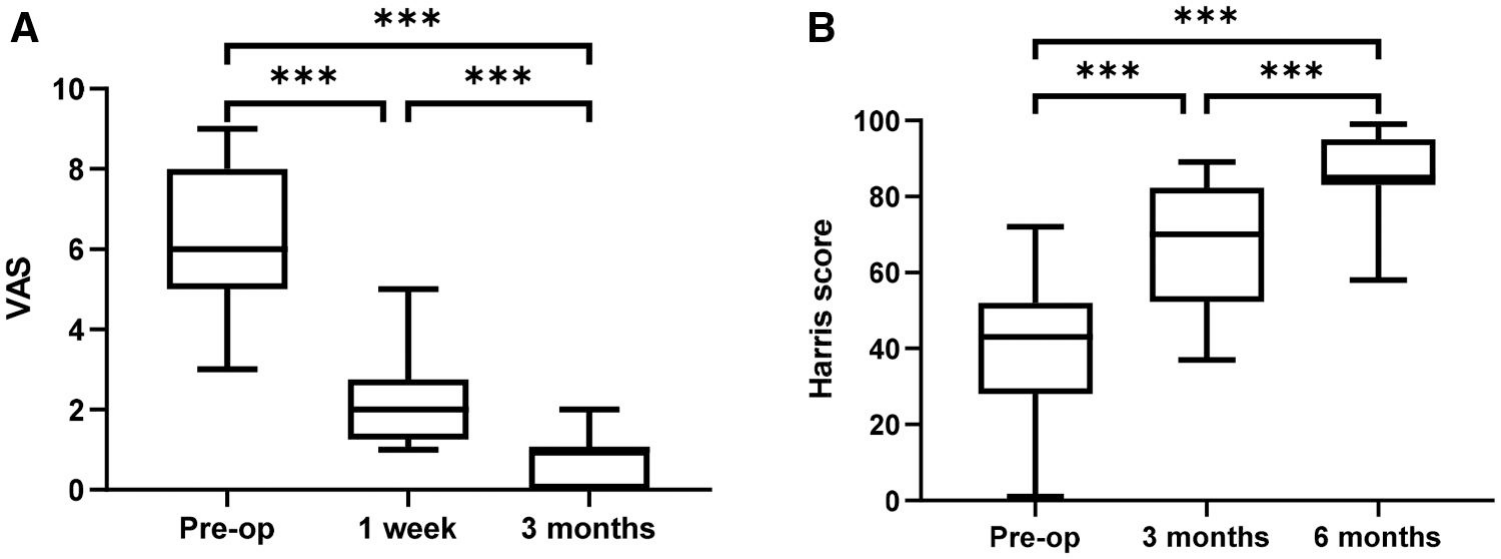
(**A**) The VAS score of 33 patients at 1 week and 3 months after the operation and (**B**) the Harris score of 33 patients at 3 months and 6 months after the operation.

A total of 33 patients underwent the surgical procedure successfully. Detailed patient demographics and clinical characteristics are presented in Table 4. Intraoperative blood loss ranged from 50 ml to 1800 ml, with a mean volume of 634 ml ± 570 ml. As illustrated in Figure 8A, the VAS scores for all 33 patients at 1 week and 3 months post-surgery were significantly lower compared to baseline scores (*P* < 0.001). Similarly, Figure 8B demonstrates that Harris scores for all patients at 3 and 6 months post-surgery were significantly higher than baseline values (*P* < 0.001). By 6 months post-surgery, all patients reported minimal to no pain. Furthermore, radiographic evaluation at postoperative day 1, 3 months and 6 months revealed stable prosthetic implants without loosening or osteolysis. 11 patients with infections were cured with one-stage surgery and all inflammatory indicators returned to normal after 3 months. Sixteen patients with bone defects had successful repairs with integrated prostheses, with no loosening observed at the last follow-up. A patient with fibrous dysplasia had their femoral deformity treated with a prosthesis during THA. None of the 33 patients experienced complications such as infection, deep vein thrombosis or wound nonunion.

**Table 4.** Patients list

Patient	Gender	Age	Diagnosis	Surgery	Function of porous prosthesis
1	M	65	PJI	RTHA	Drug delivery
2	M	69	Fibrous dysplasia	THA	Deformity correction
3	M	56	PJI	RTHA	Drug delivery
4	F	68	DDH	THA	Bone defect repairing
5	M	44	Suppurative arthritis	THA	Drug delivery
6	M	54	Hip chondrosarcoma	THA	Pelvis reconstruction
7	F	59	Acetabulum old fracture	THA	Bone defect repairing
8	M	47	Suppurative arthritis	THA	Drug delivery
9	M	67	Suppurative arthritis	THA	Drug delivery
10	M	79	Traumatic ONFH	RTHA	Bone defect repairing
11	F	57	Aseptic cup loosening	RTHA	Bone defect repairing
12	M	72	Pelvic discontinuities	THA	Pelvis reconstruction
13	M	63	Suppurative arthritis	THA	Drug delivery
14	F	44	DDH	THA	Bone defect repairing
15	M	67	PJI	RTHA	Drug delivery
16	F	43	DDH	THA	Bone defect repairing
17	M	61	PJI	RTHA	Drug delivery
18	M	63	Innominatum tumor	THA	Pelvis reconstruction
19	F	70	Tuberculosis hip	THA	Drug delivery
20	F	56	Aseptic cup loosening	RTHA	Bone defect repairing
21	F	49	DDH	THA	Pelvis reconstruction
22	M	72	Aseptic cup loosening	RTHA	Bone defect repairing
23	M	70	Aseptic stem loosening	RTHA	Bone defect repairing
24	F	66	Suppurative arthritis	THA	Drug delivery
25	F	53	Aseptic stem loosening	RTHA	Bone defect repairing
26	M	33	PJI	RTHA	Drug delivery
27	M	53	Aseptic stem loosening	RTHA	Bone defect repairing
28	F	75	OA	THA	Bone defect repairing
29	F	70	Aseptic cup loosening	RTHA	Pelvis reconstruction
30	M	66	ONFH	THA	Bone defect repairing
31	F	54	DDH	THA	Bone defect repairing
32	F	55	DDH	THA	Bone defect repairing
33	F	74	DDH	THA	Bone defect repairing

OA, osteoarthritis; ONFH: osteonecrosis of the femoral head; RTHA: revision of total hip arthroplasty.

#### Typical cases 

**Figure 9.**
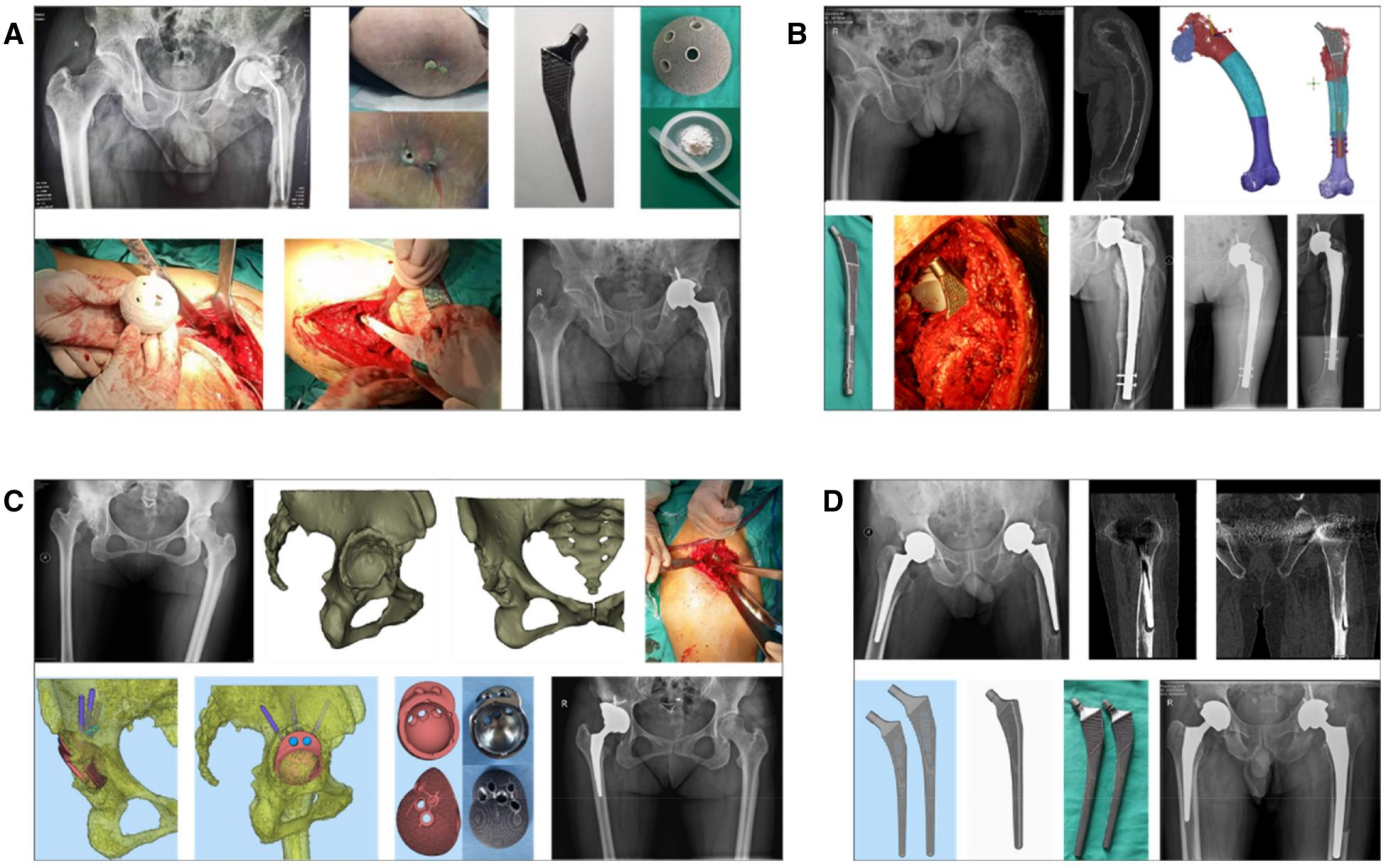
(**A**) The patient underwent revision surgery for periprosthetic infection after hip arthroplasty. A 3D-printed porous tantalum prosthesis loaded with antibiotics was used for treatment, and there was no recurrence of infection during the 30 months of follow-up. (**B**) One case of fibrous dysplasia of the proximal femur involving the hip joint was treated with an osteotomy and fixed with a custom-made femoral stem prosthesis. The patient recovered satisfactorily after 24 months of follow-up. (**C**) For osteoarthritis secondary to right developmental dysplasia of the hip, the integrated acetabular cup was used for joint replacement and the rotation center and limb length were good. (**D**) For patients with loosening of hip prosthesis after total hip arthroplasty, the use of extended 3D printed porous tantalum prosthesis can achieve satisfactory results.

Case 1 (Figure 9A): A 54-year-old man presented with an infection 10 months after hip replacement. The prosthesis was removed, and a bone cement spacer was placed. After removal of the prosthesis and thorough debridement, the SLM porous Ta prosthesis was loaded with calcium phosphate cement containing sensitive antibiotics, which was implanted into the patient's body to form a long-term stable release of antibacterial environment. The incision healed well after revision surgery, and the inflammatory biomarkers gradually decreased. No recurrence was observed during the 2-year follow-up period.

Case 2 (Figure 9B): A 45-year-old female presented with claudication for more than 30 years and pain in the left hip for 5 years. The left hip was diagnosed as left developmental dysplasia of the hip (Crowe III). The left femoral head was subluxation, and the left lower limb was 3 cm shorter than the contralateral side. A defect in the superolateral part of the acetabulum would be encountered if the center of rotation of the left hip was moved downwards as in traditional joint replacement. A structural bone graft or an acetabular augment would be necessary to reconstruct the superior wall. A customized implant containing the cup and the augment as an integral was designed and fabricated by SLM. The prosthesis was accurately implanted according to the preoperative design, filling the defect and achieving complete contact with the bone. Subsequent X-rays confirmed a return to normal limb length and offset.

Case 3 (Figure 9C): A 67-year-old man presented with left hip pain and limited mobility for 19 years. He was diagnosed with fibrous dysplasia of the left proximal femur and osteoarthritis of the left hip. The middle and lower third of the bone was affected by fibrous dysplasia, resulting in severe damage to the hip joint. Additionally, the external arch of the left femur exhibited deformities. The external arch of the left femur was deformed. The left femur correction and left hip replacement were required. Using preoperative simulation, two osteotomies were planned, and the distal end was locked after fixation with a 3D-printed femoral stem. The operation was successful, and the patient recovered well after the operation. The left hip joint function was satisfactory during the 20-month follow-up.

Case 4 (Figure 9D): A 71-year-old male patient underwent bilateral total hip arthroplasty due to bilateral osteonecrosis of the femoral head 11 years ago. The patient recovered well after surgery. Over the past 5 years, the discomfort in the left thigh progressively intensified, leading to severe restrictions in mobility. An imaging examination in the outpatient department showed that the femoral prosthesis was loose and displaced distally, and the distal part penetrated the posterolateral cortex of the middle part of the femoral shaft. We designed a distal lengthening stem that spanned the cortical defect and rested on the distal healthy bone. The prosthesis was successfully implanted, and the initial stability was good. During the follow-up, there was no prosthesis displacement, fracture or periprosthetic fracture.

## Discussion

Over the years, traditional total hip replacement has achieved satisfactory results in the repair and reconstruction of hip joint trauma, developmental dysplasia of the hip (DDH), femoral head necrosis and other injuries [21]. However, several challenges persist, including limitations in material properties, biocompatibility, biomechanics and the potential for inaccurate prosthesis implantation. These factors can contribute to the clinical occurrence of serious complications such as periprosthetic fracture secondary to stress shielding, prosthesis fracture, subsidence and infection [22]. This study sought to develop a porous Ta hip prosthesis with biological internal fixation through SLM technology to solve these intractable problems. We validated the feasibility of SLM porous Ta hip prosthesis in total hip arthroplasty from the perspective of material properties, biocompatibility and biomechanical safety. The chemical composition (especially oxygen content) and mechanical properties of SLM Ta meet the requirements of ISO 13782 for pure Ta for surgical implants. Given the widespread use of Ti6Al4V as a material for 3D-printed prostheses [23], we selected it as the control material for comparison. Biocompatibility test results showed that SLM Ta has better biosafety than SLM Ti6Al4V. Furthermore, SLM Ta could reduce the risk of inflammation following implant implantation, which helps reduce the probability of aseptic loosening and improve the long-term stability of the prosthesis. The topological optimization design was used to optimize the structure of the femoral stem while reducing its weight and maintaining sufficient biomechanical properties. The mechanical properties of the optimized hip prosthesis were confirmed to meet the clinical implantation requirements through mechanical testing. On this basis, a series of clinical trials of Ta hip prosthesis with a customized structure was conducted to verify their feasibility and advantages in treating refractory hip diseases such as reconstructive, malformation and infection. The SLM Ta hip prosthesis is expected to open up a new field in the hip prosthesis market due to its personalized characteristics, good biocompatibility, bone bioactivity and the advantages of anti-infective drugs [24].

It is now understood that the oxygen content greatly impacts the mechanical properties of SLM Ta [25]. Excessive oxygen content can lead to poor plasticity of SLM Ta. When oxygen content was below 300 ppm, the fracture elongation of SLM Ta exceeded 30%, which greatly improved the toughness of SLM Ta [25]. This investigation employed the selective laser melting (SLM) process within a high-purity argon atmosphere (99.99%). The oxygen concentration within the printing chamber remained consistently below 100 ppm throughout the process. Analysis revealed a minimal increase of only 20 ppm in the oxygen content of SLM-fabricated Ta cubes compared to the raw Ta powder. This finding suggests that the high-purity argon gas protective environment effectively mitigated the rise in oxygen content during SLM processing. The relative density and grain size of SLM-fabricated Ta are known to significantly influence its mechanical properties [26]. In this study, the relative density of SLM Ta reached 99.5%. Due to the rapid cooling of molten Ta in the printing process, the SLM Ta exhibited a fine-grained metallographic morphology, ranging from 60 μm to 100 μm, which improved the mechanical strength of SLM Ta through grain boundary strengthening. Furthermore, the high relative density of SLM Ta indicated a low occurrence of defects, resulting in a high fatigue resistance of the material [27]. Mechanical testing confirmed that SLM Ta possesses excellent overall mechanical properties, with significantly higher tensile strength and elongation compared to as-cast and wrought Ta [28]. These results highlight the unique benefits of SLM as a novel manufacturing technology.

Building on prior evidence for Ta's biocompatibility [29–31], this study confirmed good biocompatibility and lack of cytotoxicity for SLM-fabricated Ta. Furthermore, MC3T3-E1 cell proliferation on both SLM solid and porous Ta surfaces surpassed that observed on Ti6Al4V, aligning with previous reports [32–34]. SLM Ta showed higher cell adhesion of MC3T3-E1 cells compared to SLM Ti6Al4V. Furthermore, MC3T3-E1 cells proliferated rapidly and underwent osteogenic differentiation on SLM Ta, indicating a potential for better osseointegration of SLM Ta implants. Derek Avery *et al*. found that the chemical composition of the biomaterials [35] largely drives the inflammatory response. *In vitro* macrophage studies showed that SLM Ta could regulate the inflammatory microenvironment and reduce the inflammatory response caused by the implant. Chronic inflammation is one of the main causes of the aseptic loosening of the implant. After the implants were implanted into the human body, a series of biological reactions took place, including inflammatory reactions [36]. Inflammation is a protective response of the human body to injury and infection. The chemotaxis and polarization of macrophages and the release of inflammatory mediators are important manifestations of an inflammatory response [37]. It has been established that macrophages are triggered upon contact with surrounding tissue and differentiate into various subtypes. If macrophages are polarized towards the classical activation subtype (M1), it may result in elevated levels of oxidative stress and the release of inflammatory cytokines, potentially leading to chronic inflammatory response and tissue damage, which can affect the biocompatibility of the implant. Conversely, if macrophages tend to polarize towards the alternatively activated subtype (M2), they will release anti-inflammatory factors and cytokines, thereby reducing the inflammatory response and avoiding oxidative stress [38, 39].

We demonstrated that the SLM Ta implant had superior biocompatibility and caused less impact on the body, indicating a strong correlation between the SLM Ta implant, inflammation and macrophage polarization [40]. Macrophage polarization is one of the key factors affecting the biocompatibility of implants. This study revealed that Ta, compared to Ti6Al4V, significantly promoted the polarization of macrophages towards the M2 phenotype and concurrently suppressed the release of inflammatory mediators, including CCL3, CXCL2, TNF-α and ICAM-1. Notably, CCL3 and CXCL2 are well-established chemokines that play critical roles in inflammatory processes. They can recruit and activate inflammatory cells and promote the occurrence of inflammatory reactions [41]. TNF-α is a cytokine that can induce the aggregation and proliferation of inflammatory cells and promote inflammatory reactions [42]. ICAM-1 is an adhesion molecule that promotes the adhesion and migration of inflammatory cells. During the inflammation process, the expression of ICAM-1 is upregulated, thus, increasing the possibility of inflammatory cells gathering at the site of inflammation [43]. Hence, CCL3, CXCL2, TNF-α and ICAM-1 are implicated in inflammation due to their diverse roles in initiating and promoting the inflammatory cascade.

KEGG pathway analysis revealed downregulation of the TNF and chemokine signaling pathways in the SLM Ta group. This aligns with the cytokine array data, showing downregulated expression of TNF-α and CXCL2 genes within the TNF signaling pathway. It has been reported that the TNF signaling pathway can activate macrophages to produce CXCL2, thereby attracting monocytes and other immune cells into the inflammatory site and participating in the occurrence and development of the inflammatory response. In addition, TNF can activate various inflammatory response genes through the NF-κB pathway, thereby enhancing the ability of macrophages to respond to exogenous stimuli and further polarizing them into M1 macrophages. After the polarization of M1 macrophages, TNF can maintain their polarization and further promote the activation of M1 macrophages and the occurrence and maintenance of inflammatory response [44]. In the chemokine signaling pathway, the expression of CCL3 and CXCL2 genes was significantly downregulated, consistent with the results of cytokine array analysis. Two Chemokines, CCL3 and CXCL2, also play an important role in macrophage polarization and inflammatory response. For example, CCL3 was found to induce macrophage polarization toward M1 macrophages. M1 polarization of macrophages triggers CCL3-mediated inflammatory and apoptotic responses [45]. CXCL2 can lead to the polarization of macrophages to M1 macrophages by activating the MyD88/TRIF pathway and regulating the inflammatory response [46].

Besides, we found that the TNF signaling pathway and Chemokine signaling pathway of macrophages were significantly downregulated in the SLM Ta group compared to the SLM Ti6Al4V group. This downregulation essentially inhibits other immune cells from aggregating at inflammatory sites and reduces the activation of inflammatory function in immune cells. These results indicate that SLM Ta exhibits lower immunogenicity and a higher capacity for M2 polarization induction of macrophages. The immune affinity properties exhibited by the SLM Ta joint prosthesis may contribute to its *in vivo* biocompatibility and osseointegration. Therefore, the *in vitro* cell test verified that Ta, as a medical metal material, has superior biological properties for bone formation compared to SLM Ti6Al4V. This conclusion is supported by the literature. Derek Avery *et al*. conducted *in vivo* and *in vitro* experiments to compare the inflammatory response of pure titanium, Ti6Al4V, PEEK and 316 L stainless steel. Their findings also confirm that the chemical composition of materials plays a significant role in immune cell response [35].

This investigation was undertaken to promote biological osseointegration between the joint prosthesis and surrounding bone tissue. This objective was achieved by encouraging bone ingrowth into the porous Ta structure. The implemented porous design offers the additional benefits of reduced weight and stiffness for the prosthesis. To accomplish these goals, the structure of the SLM-fabricated Ta hip prosthesis was optimized using topological design principles. Furthermore, the lightweight design was meticulously tailored to ensure that it could withstand the requisite biomechanical loads experienced during *in vivo* function within the human body. It is well-established that fatigue resistance of the femoral stem is paramount for guaranteeing the mechanical safety of the implant during its *in vivo* service life [47]. According to the mechanical test results, the SLM Ta femoral stem can endure long-term, periodic physiological loads *in vivo* despite its lightweight design and is not susceptible to fatigue-induced fractures. The porous structure of the SLM Ta joint prosthesis is expected to reduce stress shielding and avoid bone resorption around the prosthesis. Arabnejad *et al*. [14] demonstrated that a fully porous implant with an optimized material microstructure could reduce the amount of bone loss secondary to stress shielding by 75% compared to a fully solid implant. Conversely, the bone tissue around the prosthesis can form a mechanical lock with porous Ta; thus, a long-term stable osteointegration between the prosthesis and its surrounding bone tissue can be achieved [47].

In the clinical application of SLM porous Ta hip protheses, there were no cases of infection or prosthesis loosening, and even in cases of prosthetic joint infection (PJI), the infection was effectively controlled without recurrence, indicating that the prosthesis is compatible with human tissue and can reduce inflammation. Traditional treatment of PJI typically involves two or more surgeries, with infection treatment followed by revision surgery. However, in this study, infection treatment and prosthesis revision were accomplished in a single surgery, suggesting that prosthesis may be crucial in reducing the inflammatory response.

The current study is limited by a follow-up period of only four years for patients implanted with SLM porous Ta hip prostheses. This timeframe is insufficient to definitively assess the long-term service life of the prosthesis. Ta's high inherent density presents a challenge in accurately evaluating bone ingrowth and periprosthetic bone volume using X-ray imaging techniques.

## Conclusions

Ta offers significant advantages as an orthopedic implant material in clinical settings. Additive manufacturing technology has unlocked promising avenues for the clinical use of Ta in orthopedic implants. Macrophage cell assays demonstrate that SLM Ta exhibits lower immunogenicity and a greater capacity for inducing macrophage M2 polarization. Following the principles of biological fixation, the SLM Ta hip prosthesis was designed with an optimized topological structure to meet the biomechanical demands of the human body while enabling personalized anatomical matching. Additionally, the prosthesis boasts the capability to deliver anti-infective drugs to surrounding tissue, further enhancing its functionality. Postoperative follow-up over 1–2 years has revealed a positive prognosis and satisfactory restoration of joint function in patients implanted with the SLM hip prosthesis. The convergence of additive manufacturing and medical technologies presents a promising future for Ta as an orthopedic implant material, offering solutions to the challenges of hip joint replacement.

## Data Availability

This paper presents all data required for the evaluation of its conclusions. Requests for further information related to this work can be directed to the corresponding author.
